# Epigenome Aberrations: Emerging Driving Factors of the Clear Cell Renal Cell Carcinoma

**DOI:** 10.3390/ijms18081774

**Published:** 2017-08-16

**Authors:** Ali Mehdi, Yasser Riazalhosseini

**Affiliations:** 1 Department of Human Genetics, McGill University, 1205 Dr Penfield Avenue, Montreal, QC H3A 1B1, Canada; ali.mehdi@mail.mcgill.ca; 2 McGill University and Genome Quebec Innovation Centre, 740 Dr Penfield Avenue, Montreal, QC H3A 0G1, Canada

**Keywords:** renal cell carcinoma, DNA methylation, histone modifications, lncRNA, HIF, VHL, ccRCC, epigenetic therapy

## Abstract

Clear cell renal cell carcinoma (ccRCC), the most common form of Kidney cancer, is characterized by frequent mutations of the von Hippel-Lindau (*VHL*) tumor suppressor gene in ~85% of sporadic cases. Loss of pVHL function affects multiple cellular processes, among which the activation of hypoxia inducible factor (HIF) pathway is the best-known function. Constitutive activation of HIF signaling in turn activates hundreds of genes involved in numerous oncogenic pathways, which contribute to the development or progression of ccRCC. Although *VHL* mutations are considered as drivers of ccRCC, they are not sufficient to cause the disease. Recent genome-wide sequencing studies of ccRCC have revealed that mutations of genes coding for epigenome modifiers and chromatin remodelers, including *PBRM1*, *SETD2* and *BAP1*, are the most common somatic genetic abnormalities after *VHL* mutations in these tumors. Moreover, recent research has shed light on the extent of abnormal epigenome alterations in ccRCC tumors, including aberrant DNA methylation patterns, abnormal histone modifications and deregulated expression of non-coding RNAs. In this review, we discuss the epigenetic modifiers that are commonly mutated in ccRCC, and our growing knowledge of the cellular processes that are impacted by them. Furthermore, we explore new avenues for developing therapeutic approaches based on our knowledge of epigenome aberrations of ccRCC.

## 1. Introduction

Kidney cancers account for the 7th and 10th most common malignancies in males and females, respectively, in the US according to estimated new cases in 2014 [1]. Renal cell carcinoma (RCC) is the most common form (85–90% of all kidney cancers) and contributes to 2.4–4% of all human cancers [1,2,3,4,5]. More than 300,000 patients are diagnosed each year and around 143,000 of them die each year, representing RCC as the sixteenth most common cause of death from cancer worldwide [2]. RCC manifests in different histological types, of which clear cell RCC (ccRCC) is the most common with a prevalence of 70–80%; followed by papillary RCC (10–16%); chromophobe RCC (5–7%); and other RCCs types with lower prevalence [6,7]. Risk factors for the development of RCC include smoking tobacco [8], obesity [9,10], hypertension [11] and a history of chronic kidney disease [12]. Furthermore, RCC has also been identified as an occupational disease as exposure to certain chemicals such as trichloroethylene [13], which was confirmed in a meta-analysis [14,15,16], elevates the risk of RCC. Moreover, exposure to cadmium and cadmium compounds has also been reported as a potential factor that increases risk of RCC [13]. In addition, genomic studies have indicated that an exposure to aristolochic acid (a nephrotoxin) may have a possible role in the tumorigenesis of the ccRCC [17,18]. Other risk factors include radiation exposure (e.g., for cancer treatment) that slightly increases the risk and rare hereditary conditions such as von Hippel-Lindau disease, caused by germline mutations in *VHL* gene [2]. Furthermore, an unexplained male predominance exists in RCC with a male: female incidence rate of 1.5:1–2:1 in Europe and the USA [1,2,19,20,21,22]. The differences between male and female prevalence across the world remain valid even after correcting for the confounding variables such as gross domestic product, geographical region, and environmental risk factors, including tobacco exposure [4,21].

ccRCC arises from the epithelium of the proximal tubule of the nephron of kidney [6,7,23], and exhibits a distinct histology where each cell appears to have a round nucleus within a clear cytoplasm, and is separated from neighboring cells by a distinct cell membrane [23]. Although most ccRCCs are treated by resection via surgery, early diagnosis is difficult and by the time the ccRCC is detected, it has already metastasized in 30–35% of the patients [5,24]. In fact, there is no recommended screening test for the early detection of RCC among people at average risk [2]. Even though patients present with localized disease (stages I or II) have a 5-year survival rate greater than 70% with radical or partial nephrectomy, stage III (regional spread) and IV (metastatic) RCC have a poor prognosis, with 5-year survival rates of only 50% and 10%, respectively [2,25,26]. Metastatic RCC (mRCC) is resistant to chemotherapy and radiotherapies and is incurable [7]. Therefore, medical researchers hope that increasing knowledge of the molecular biology of tumor initiation, development and metastasis will help to develop better diagnostic and therapeutic tools.

Following the advent of next generation sequencing (NGS), RCC was of the first tumor types whose genomes were sequenced using massively parallel sequencing [6]. Exome sequencing was performed in a combined effort to determine protein coding genes that were affected by somatic mutations [27,28,29]. Strikingly, numerous genes that are involved in the regulation of epigenetic modifications were discovered to be commonly mutated in RCC tumors. This pointed to the notion that abnormalities of epigenetic modifiers are likely among the key molecular driving factors of RCC. Since then additional studies including the Cancer Genome Atlas (TCGA) [30] and the International Cancer Genome Consortium projects [18]. Studies have confirmed frequent mutations of the histone modifying as well as chromatin remodeler genes in RCC [30,31].

Epigenetic factors including DNA methylation patterns and histone modifications have a central role in the regulation of global and local gene expression [32,33]. Deregulation of these epigenetic regulatory mechanisms is involved in tumorigenesis of different cancers including RCC [32,33]. Here, we review genomic and epigenomic abnormalities that drive RCC, with an emphasis on the recently-recognized frequent aberrations of epigenetic regulators. First, role of the well-known Von Hippel-Lindau (VHL)-Hypoxia-inducible factors (HIF) pathway will be briefly mentioned. Next, we will review epigenetic modifier genes that are recurrently found mutated in recent RCC genomics studies, and will discuss the emerging knowledge about functional consequences of their mutations, and how these abnormalities may contribute to RCC development. Next, a connection between these genes and aberrant DNA methylation, histone modification and long non-coding RNA (lncRNA) expression patterns in RCC will be discussed. Finally, we will examine the clinical significance of epigenome abnormalities in RCC. The epigenetic regulations mediated by miRNA are not the focus of this review as they have extensively been reviewed elsewhere [6,34,35].

## 2. Von Hippel-Lindau (VHL) and Hypoxia-Inducible Factors (HIF)

In contrast to other cancers, inactivating mutations in general tumor suppressor genes (TSGs), such as *TP53* and *RB1*, are not frequent in RCC. In fact, mutations in *TP53* and *RB1* are found only in 11% and <1% in all kidney cancers, respectively; while another TSG, *CDKN2A*, is mutated in only 10% of RCC tumors [6,36]. On the other hand, ccRCC has a distinct genetic background as we discuss below.

Germline mutations in von Hippel-Lindau (*VHL*) tumor suppressor gene result in autosomal dominant von Hippel Lindau syndrome and predispose to ccRCC [6,7,23,37,38,39,40,41,42]. VHL disease is an inherited disorder that follows Knudson’s two-hit tumor suppressor model [23,43,44,45,46]. Tumors associated with VHL disease include ccRCC (2–4% of all cases), blood tumors (hemangioblastomas) of the central nervous system and retina, adrenal gland tumors (pheochromocytoma), and pancreatic and inner ear tumors [23,38,39,41,42,47]. The typical renal manifestations are kidney cysts and ccRCC [23,38,41,47]. In sporadic (non-familial) form of ccRCC, *VHL* is biallelically mutated or epigenetically silenced in ~85% of tumors [48].

*VHL* is a tumor suppressor gene which is located at Chromosome 3p25 [37,41,49,50,51]. It encodes two very functionally similar proteins of ~28–30 kDa and 19 kDa, due to alternative translation initiation [38,39,52,53,54]. Majority of *VHL* mutations are inactivating events that typically disrupt function of both forms of the protein (pVHL) [30,49,55,56]. pVHL is a multi-functional factor that acts as an adaptor protein recruiting different effector proteins to different targets thereby regulating various cellular processes [23,41,50]. These cellular processes include glucose uptake and metabolism, angiogenesis [6,57], suppressing epithelial to mesenchymal transition (EMT) [24,58], cellular senescence [59], activating p53 [60], pH homeostasis, chemotaxis, proliferation, survival, apoptosis [6,57,61], transcription regulation [6], preventing aneuploidy [62], secreting components of extracellular matrix [63,64], controlling internalization of growth factor receptor [65] regulating the canonical WNT signaling [66], ubiquitinating RNA polymerase II [67] degrading β2-adrenergic receptors [68], and regulating nuclear factor (NF) κB activity [69]. However, pVHL is best known as a regulator of oxygen and energy sensing by targeting hypoxia-inducible factors (HIF) 1 and HIF2 for proteasomal degradation, a process essential in regulating hypoxia-driven gene expression [6,23,38,41,47,50,57]. Specifically, pVHL forms a complex with Elongin B, Elongin C, Cul2, and Rbx1 proteins that functions as an E3 ubiquitin ligase that ubquitylates HIF subunits for proteasomal degradation [41,50].

HIFs are transcription factors of mainly five types: HIF1α, HIF2α, HIF3α, HIF1β (or ARNT1 (aryl hydrocarbon nuclear translocators 1)) and HIF2β (or ARNT2) [23,38,41,47,50,70]. HIF1α and HIF2α are encoded by *HIF1A* and *EPAS1*, respectively. HIF1α is constitutively expressed while HIF2α expression is restricted [23,38,41,47,50]. HIFα and HIFβ proteins form a complex, which translocates to the nucleus where it binds to DNA at hypoxia-response elements (HREs) through two basic helix–loop–helix (bHLH) domains of HIFα, and subsequently activates transcription of hypoxia-inducible genes. HIFs regulate hundreds of genes related to acute or chronic adaptation to hypoxia. Furthermore, HIFs regulate genes that function in glucose uptake and metabolism, control of extracellular pH, erythropoiesis and angiogenesis, mitogenesis and apoptosis [23,38,41,47,50].

### 2.1. Physiology of the VHL-HIF Pathway

For pVHL to recognize HIFα, HIFα must be hydroxylated. HIFα hydroxylation happens under the normoxic conditions, in the presence of oxygen, by an enzyme prolyl hydroxylase (PHD or EglN) that requires iron, ascorbate and 2-oxoglutarate [38,41,50,70,71,72,73]. EglN, especially EglN1, uses oxygen to add hydroxyl group on one of two conserved proline residues in the Oxygen Degradation Domain of the α-subunit of the HIFα molecule [70,74,75]. This allows pVHL to recognize the hydroxylated HIFα, subsequently ubiquitinylate it for proteasomal degradation [23,38,39,41,42,47,50,57]. However, under low oxygen levels, hydroxylation step by EglN does not happen, resulting in HIFα stabilization due to the lack of recognition by pVHL. HIFα accumulates in the cytoplasm, forms complex with constitutionally expressed HIFβ, and eventually translocates into the nucleus and binds to the HREs, thereby activating hundreds of genes related to adaptation to a low oxygen environment [7,23,38,39,41,42,50].

### 2.2. Hypoxia-Driven Tumorigenesis of ccRCC

**Figure 1:**
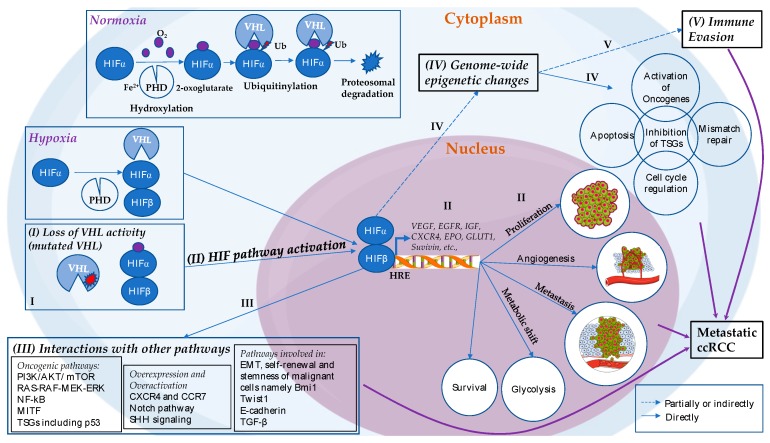
Physiology of the VHL-HIF pathway along with proposed etiopathogenesis that leads to metastatic ccRCC. In normoxia, HIFα hydroxylation is carried out by PHDs that requires iron, ascorbate and 2-oxoglutarate. PHD proteins use oxygen to add hydroxyl group on one of two conserved proline residues in the Oxygen Degradation Domain of the α-subunit of the HIFα molecule in order to mark it for recognition by pVHL. pVHL then recognizes hydroxylated HIFα, ubiquitinylates it and targets it for proteasomal degradation. However, in hypoxia, hydroxylation step by PHDs does not happen, resulting in HIFα stabilization. HIFα accumulates in the cytoplasm, forms complex with constitutionally expressed HIFβ, and eventually translocates into the nucleus where is binds to HREs, thereby activating hundreds of genes related to adaptation to a low oxygen environment. (**I**) Loss of VHL activity; (**II**) Constitutively active HIF pathway: When the VHL is mutated, pVHL is non-functional, resulting in stabilization of HIFα, and downstream overexpression of numerous genes involved in angiogenesis (e.g., VEGFA), proliferation (e.g., EGFR), cell migration and invasion (e.g., CXCR4), metabolic shift towards glycolysis (e.g., GLUT1), survival (e.g., survivin), erythropoiesis (e.g., EPO), which ultimately contribute to ccRCC tumorigenesis; (**III**) Interaction of the HIF pathway with other pathways: While HIF pathway interacts with and enhances the activity of other oncogenic pathways, it may also contribute to the genome-wide epigenetic alterations; (**IV**) Genome-wide epigenetic alterations: The epigenome patterns, established also due to mutations of multiple TSGs involved in epigenetic modifications, alter various oncogenic, apoptotic, cell cycle regulatory and mismatch repair pathways; and (**V**) Immune evasion: Lastly, immune evasion occurs in ccRCC which is suggested to be partially enhanced by genome-wide epigenetic alterations. All these interactions and mechanisms interplay throughout the pathogenesis and carcinogenesis of the metastatic ccRCC [42]. Abbreviations: VHL, von Hippel Lindau; HIF, hypoxia inducible factor; PHD, prolyl hydroxylase; Ub, ubiquitin; HRE, hypoxia response elements; PI3K, phosphoinositide 3-kinase; mTOR, mammalian target of rapamycin; NF-κB, nuclear factor kappa B; MITF, microphthalmia-associated transcription factor; CXCR4, CXC chemokine receptor 4; CCR7, C-C chemokine receptor type 7; SHH, sonic hedgehog; TSGs, tumor suppressor genes, EMT, epithelial to mesenchymal transition; TGF-β, transforming growth factor beta; and ccRCC, clear cell renal cell carcinoma.

An etiopathogenesis of metastatic ccRCC was proposed recently describing putative stepwise molecular alterations that lead to the initiation and progression of the disease, focusing on the role of VHL-HIF molecular axis (Figure 1) [42]. This process starts with inactivation of pVHL by mutations or epigenetic silencing of the *VHL* gene. As such, even though HIFα is hydroxylated, pVHL is non-functional, resulting in the constitutive activation of HIFα. HIFα is stabilized, dimerizes with HIF1β and activates numerous genes involved in angiogenesis (e.g., *VEGFA*), proliferation (e.g., *EGFR*), cell migration and invasion (e.g., *CXCR4*), metabolic shift towards glycolysis (e.g., *GLUT1*), survival (e.g., *survivin*), and erythropoiesis (e.g., *EPO*), which ultimately contribute to tumorigenesis [7,23,41,42,50]. Whereas there is evidence that HIF-2α is oncogenic, an oncogenic role for HIF-1α is not well-documented, and the two proteins appear to show contrasting functions in VHL-deficient cells [23,38,39,50,76,77,78,79,80]. The constitutively active HIFα pathway is considered as a key driving pathway of ccRCC [42], due to its broad actions on activating multiple downstream oncogenic pathways and its potential involvement in establishing abnormal epigenome patterns as depicted in Figure 1. Recently, two antagonists of HIF-2α (PT2385 and PT2399) have shown promising results in inhibiting tumor growth and angiogenesis in ccRCC [40,81,82,83] highlighting HIF-2α as potential therapeutic target. However, given the wide spectrum of cellular processes that can be regulated by pVHL, the consequences of VHL-deficiency are beyond the activation of HIF signaling, pointing to the diverse molecular mechanisms that are deregulated in ccRCC, and can potentially serve as therapeutic targets [84].

## 3. Epigenetics of ccRCC

Epigenetics refers to the heritable changes that govern gene expression patterns not by affecting the sequence of DNA, but through modulating structure of the DNA [85,86,87]. Abnormal epigenome patterns play a central role in dysregulation of gene expression in cancer, and are therefore considered as putative drivers of tumorigenesis and a hallmark of cancer [88,89]. However, among vital questions to be answered in this area are what are the drivers of epigenome aberrations and how can epigenome abnormalities cause cancer? Here we review the current knowledge about mutational status of genes coding for epigenome modifier proteins in RCC, and discuss their emerging contributions to the RCC-associated epigenome alterations including changes in histone modifications, DNA methylation and lncRNAs expression, and downstream consequences in the pathogenesis of RCC.

### 3.1. Histone Modifications

In eukaryotes, the DNA is packed in a form of a series of “beads on a string” where the string is the DNA and each bead is a nucleosome core particle consisting of approximately 146 bp of DNA molecule, which is wrapped around an octamer of histone proteins (two of each: H2A, H2B, H3 and H4) [90]. The DNA, nucleosomes and some non-histone proteins such as high mobility group (HMG) proteins together form the chromatin. Chromatin can be either relaxed (euchromatin) or condensed (heterochromatin). Generally, euchromatin is the state where genes are transcriptionally active as the transcriptional machinery can access the DNA, whereas transcription of genes located at heterochromatic regions is silenced [6,90,91]. The changes in the chromatin structure are influenced by chemical modifications of amino acids in the external tails of histone molecules. For example, lysine residues can undergo acetylation, methylation or ubiquitylation. Likewise, arginine residues can be methylated and the serine residues can be phosphorylated [90]. These histone modifications are carried out by enzymes, namely histone acetyltransferases (HATs); histone deacetylases (HDACs); histone methytransferases (HMTs); and histone demethylases (HDMTs). HATs and HDAC add or remove acetyl groups while HMTs and HDMT add or remove methyl groups, respectively [6,35,90]. The combination of these chemical modifications forms the “histone code”, which regulates changes in the chromatin state, which in turn regulates the gene expression [6]. For instance, generally acetylation by HATs results in transcriptional activation and is more associated with a relaxed or euchromatin conformation [6,90,91]. In contrast, methylation depends upon the degree of methylation (mono-, di- or tri-methylation) and the residue affected. For instance, methylation of H3K4me3, and H3K36me3 is associated with transcriptional activation while H3K9me2, and H3K27me3 are linked to transcriptional repression [35,92]. In the following section, we discuss the recent genomics data about abnormalities of genes coding for histone and chromatin modifiers and their contributions to the ccRCC tumorigenesis.

#### 3.1.1. Chromosome 3p: A Hub for Mutated Epigenome Modifiers

**Figure 2:**
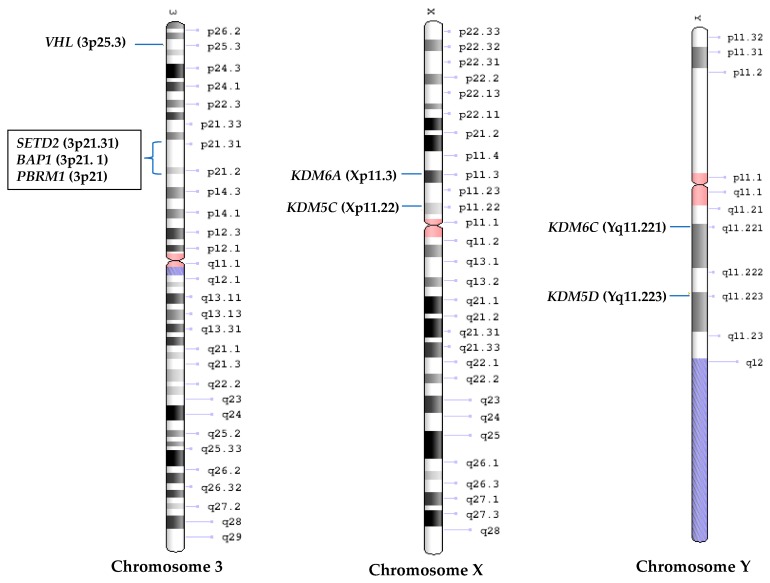
Location of frequently mutated or downregulated epigenome modifier genes including *PBRM1*, *BAP1*, *SETD2*, *KDM5C* and *KDM6A* in ccRCC. Chromosome 3p contains a ~50 Mbs region where four of these TSGs are located next to each other. Moreover, *KDM5C* and *KDM6A* are located on X chromosome but escape X-inactivation (EXITS). *KDM5D* and *KDM6C*, that are located on the Y chromosome, are homologs of *KDM5C* and *KDM6A* respectively. Interestingly, these chromosome Y homologs of the X-lined genes are commonly deleted in male patients affected by ccRCC. Figure not drawn to scale. Pink, Centromere; Black, G-positive band; White, G-negative band; Gray, G-positive band (with increasing gray color intensity indicating increasing positive band 25–75%); and Purple, variable region.

There is a long latency (>30 years) for *VHL* mutated patients to develop ccRCC [47,93] and *Vhl* deficient mice are incapable of developing ccRCC [94]. Furthermore, inactivation of *VHL* gene alone is not sufficient to cause ccRCC [23,95]. All these facts suggest that there are other genes which are likely to be important in ccRCC tumorigenesis [96,97,98]. Interestingly, 90% of sporadic ccRCCs are affected by a deletion of ~50 Mb on chromosome 3p, where besides *VHL*, three genes whose products are involved in epigenetic or chromatic modification are located; *PBRM1*, *BAP1* and *SETD2.* The co-localization of these genes next to each other suggest that they might be functionally linked [99] (Figure 2), and that a single deletion of this region could result in loss of one copy of all four genes [100,101,102]. Interestingly, all these genes have recently been associated with the pathogenesis of cancers other than ccRCC, including melanoma, mesothelioma, pancreatic cancer, colorectal cancer, leukaemia, suggesting a potential widespread tumor suppressive function for them [103,104,105,106,107,108,109]. Below we review mutational status and emerging function of them in ccRCC.

##### Polybromo 1 (PBRM1)

*PBRM1* maps to chromosome 3p21 and is the second most frequently mutated gene in ccRCC affecting about 41% of the patient tumors [29,91]. *PBRM1* encodes BAF180 protein that is the chromatin targeting subunit of the polybromo BRG1-associated factor (PBAF), which is part of the switching defective/sucrose nonfermenting (SWI/SNF) chromatin remodelling complex [110,111]. The SWI/SNF complexes are subdivided into BRG1-associated factor (BAF) and PBAF complexes. Targeting of PBAF complexes to chromatin involves BAF180 and BAF200 [110,112]. BAF180 contains six tandem bromodomains (BDs) that bind acetylated lysines on histone tails, possibly facilitating the targeting of PBAF to chromatin. The SWI/SNF multimeric complex utilizes ATP to mobilize nucleosome and thereby modulate chromatin structure [95,111], and has essential roles in replication, transcription, DNA repair, cell death, metabolism and control of cell proliferation/differentiation [110,112]. Furthermore, this complex is a key regulator of gene expression through its association with large numbers of transcription factors [6,7].

*PBRM1* functions as a two-hit TSG and majority of mutations are truncating resulting in loss of the protein [29,113,114]. The inactivation of *PBRM1* is associated with enhanced cell proliferation and migration, and its re-expression in PBRM1-decificent RCC cells has opposite effects by inducing G1 cell cycle arrest via elevation of cyclin-dependent kinase inhibitor p21 levels [29,115,116]. In addition, *PBRM1* can module cytoskeleton and cell motility by regulating the expression of genes whose products are involved in cell-adhesion and cell-signaling (e.g., E-cadherin) [117,118]. Also, BAF180 has been suggested to promote centromeric cohesion in mouse and human cells and prevent genome instability and aneuploidy [119].

A recent study has examined the consequences of BAF180 loss in *VHL*^−/−^ ccRCC cell lines and mouse models [120]. The authors showed that proliferation of ccRCC cells was suppressed only when WT exogenous BAF180 was expressed and not with tumor-associated BAF180 mutant in A704 cells. Moreover, using biochemical and functional studies, they suggested that BAF180 ability to form canonical PBAF complex, which contains the BRG1 subunit, causes growth suppression by dampening the HIF transcriptional signature. Notably, inactivation of BAF180, due to *PBRM1* mutations, results in amplification of HIF transcriptional signature in pVHL-defective (*VHL*^−/−^) ccRCC in vitro and in vivo [120].

Emerging animal models continue to advance our knowledge about the mechanisms by which *PBRM1* deficiency contributes to RCC development. Nargund et al. via tissue-specific deletion of both *Vhl* and *Pbrm1*, created a ccRCC mouse model that recapitulates histopathological and molecular features of human ccRCC and elucidated how *PBRM1* functions as a TSG in ccRCC. They suggested that genetic deletion of *Pbrm1* in mouse kidney alone results in hydronephrosis but not in ccRCC. However, simultaneous genetic deletions of both *Vhl* and *Pbrm1* in mouse kidney resulted in polycystic kidney disease, increased mortality, and the development of multifocal and transplantable ccRCC. Importantly, the stepwise morphological progression from normal appearance through cystic changes (~6 months) to ccRCC formation (~10 months) observed in this model offered an opportunity to temporally dissect the mechanisms by which *PBRM1* loss cooperates with *VHL* loss to develop ccRCC. Gene expression profiling of renal cortices from wild-type, *Vhl*-deficient, *Pbrm1*-deficient, and *Vhl* and *Pbrm1* doubly deficient mice differentiated all groups from each other where *Pbrm1* loss was suggested to amplify the transcriptional outputs of Hif1 and Stat3 incurred by the Vhl loss. *PBRM1* acted like a transcriptional resistor to prevent uncontrolled self-perpetuating amplification of the HIF1 and STAT3 transcriptional outputs incurred by *Vhl* deficiency. In addition, analysis of mouse and human ccRCC has revealed that *VHL* and *PBRM1* doubly deficient tumors displayed a convergence on the mTOR pathway activation after a long latency period, suggesting that the activation of mTOR serves as the third oncogenic driver event in ccRCC following the loss of *VHL* and *PBRM1* [91].

##### BRCA1 Associated Protein-1 (BAP1)

*BAP1* is located on chromosome 3p between *VHL* and *PBRM1* genes [121]. *BAP1* is mutated in 10–15% of the ccRCC tumors and like *PBRM1*, its mutations result in loss of the protein [122]. Somatic and germline mutations of *BAP1* results in metastasizing uveal melanoma and mesothelioma [104,105,123,124], and a syndrome characterized by uveal, cutaneous melanoma and mesothelioma, respectively [123,125]. Like *VHL*, germline *BAP1* mutations predisposes to RCC suggesting that *BAP1* loss can initiate RCC development [121,125]. The protein encoded by *BAP1* is a deubiquitylating enzyme of the ubiquitin C-terminal hydrolase (UCH) family [126]. BAP1 localizes to the nucleus, and the nuclear localization is required for its tumor-suppressor function [127]. BAP1 forms a multi-protein complex with breast cancer type 1 susceptibility protein (BRCA1) and BRCA1-associated RING domain protein 1 (BARD1) thereby regulating various processes including response to DNA damage and cell cycle control [128]. The cell cycle regulatory function of BAP1 is mediated by host cell factor-1 (HCF-1) that serves as scaffold protein that recruits BAP1 to E2F transcription factor 1 (E2F1) responsive promoters [105,129]. Once recruited, BAP1 deubiquinates H2AK119ub1 on these promoters which results in progression of cell cycle to S phase. In contrast, BAP1 knockdown can lead to G1 arrest due to increased levels of H2AK119ub1 on E2F responsive promoters [126]. Moreover, loss of BAP1 lowers the fidelity of the G1/S phase checkpoint, thus facilitating uncontrolled cell growth [7].

**Figure 3:**
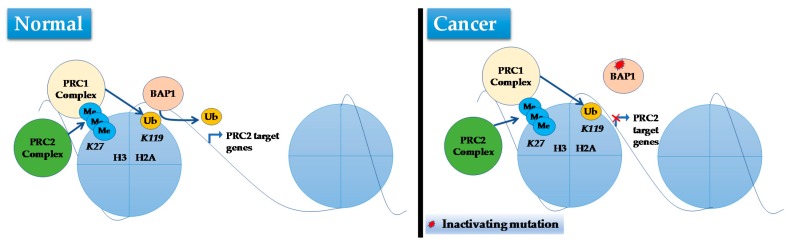
Role of BAP1 in regulation of polycomb target genes. Polycomb Repressive Complex (PRC) 2 mediates H3K27 trimethylation that triggers recruitment of PRC1 complex to H2K27me3 sites where PRC1 complex silences gene expression through monoubiquitylation of H2AK119. In normal conditions, BAP1 can contribute to the removal of ubiquitin from H2AK119ub, establishing a balance between ubiquitination and deubiquitination of H2AK119ub that regulates expression of polycomb target genes. This balance is disrupted when BAP1 is mutated in ccRCC, resulting in dysregulation of polycomb target genes.

The exact function of BAP1 in the context of RCC pathogenesis remains largely unknown but data from multiple studies converge on a role in the regulation of gene expression [104,105,130,131]. For example, pathway analyses showed epidermal growth factor (EGF), nerve growth factor (NGF), insulin and PI3K pathways were enriched with upregulated genes in *BAP1* mutant tumors [121]. Moreover, a correlation between *BAP1* mutational status in ccRCC and markers of mTORC1 activation was reported [118,122]. However, this correlation seems to be indirect as *BAP1* reintroduction into *BAP1*-deficient ccRCC cell lines did not apparently affect mTORC1 activity [119]. In addition, *BAP1* mutations in ccRCC tumors were associated with downregulated expression of target genes of polycomb repressive complex 2 (PRC2) [132] (Figure 3).

##### SET Domain Containing 2 (SETD2)

*SETD2* is also located at the chromosome 3p near the *VHL*, *PBRM1*, *BAP1* genes and is inactivated in 3–12% of RCCs [27,28,30,133]. *SETD2* encodes a H3K36 histone methytransferase [134], and its inactivation results in the global reduction of the histone mark H3K36me3 [135], and a loss of non-promoter DNA methylation across the genome [30]. Double deletion of *SETD2* in mouse models leads to loss of H3K36me3, defects in vasculature system, and embryonic lethality [136]. Loss of Setd2 function only affects H3K36 trimethylation and not di- or monomethylation of H3K36 as observed in *Setd2*^−/−^ mice.

**Figure 4:**
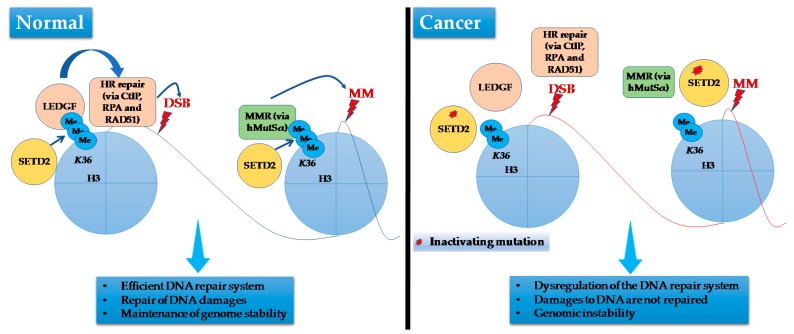
Trimethylation of H3K36 by SETD2 is required for function of DNA repair system via homologous recombination (HR) repair and mismatch repair (MMR). HR repair of DNA double-strand breaks (DSBs) occurs with the help of Lens epithelium-derived growth factor p75 (LEDGF) that binds to H3K36me3 and allows recruitment of C-terminal binding protein interacting protein (CtIP) to the DSB sites. This further allows Replication Protein A (RPA) and RAD51 binding to damaged sites. In addition, H3K36me3 contributes to MMR via recruiting the mismatch recognition protein hMutSα onto chromatin. SETD2 inactivation results in the global reduction of the histone mark H3K36me3. This leads to deficiency in DNA repair systems which in turn contributes to genome instability. Blue DNA lines means DNA is repaired while Red DNA lines means DNA is not repaired.

Recent studies have provided novel insight about the involvement of SETD2 protein in pathways connected to cancer. It has been shown that SETD2 is required for DNA mismatch repair [137], repair of DNA double-strand breaks [138], and for genome stability [139]. These functions are apparently mediated by the methyltransferase activity of SETD2 as trimethylation at H3K36 is essential for ATM and TP53-mediated DNA damage control checkpoint activation [140]. and the recruitment of the DNA mismatch repair protein Msh2 (also called hMutSα) [137] (Figure 4). Furthermore, loss of *SETD2* together with *PBRM1* mutations results in genome instability and prevents correct cell-cycle checkpoint control. ccRCCs with mutated *SETD2* show an impaired DNA-damage response [138].

The H3K36 mark is associated with open chromatin and reduced CpG methylation [135,141], hence, changes in heterochromatin structure can lead to abnormal changes in chromatin accessibility of spliceosome machinery to the genes, resulting in aberrant RNA processing and splicing [6,138,141]. Another study confirmed that monoallelic loss of *SETD2* does not alter the H3K36me3 levels as *SETD2* copy number (CN) loss occurred with high frequency (>90%) but H3K36me3 was not significantly impacted in *SETD2*^−/+^ cells [142]. Furthermore, *SETD2* mutations were suggested to correlate with decreased H3K36me3 in early-stage ccRCC and H3K36me3 was progressively dysregulated in metastasis. However, the complete loss of SETD2 function was not necessary to initiate the cancer phenotype or metastasis, and a gradual decline in H3K36me3 is due to adaptation processes or mediated by other mechanisms while developing distant metastasis. Moreover, H3K36me3 levels were not predictive of clinical site of metastasis or of the number of distant metastasis. By using ChIP-Seq and RNA-seq, authors suggested that alterations in regional H3K36me3 patterns impact on alternative splicing in ccRCC [30,133,142].

#### 3.1.2. Epigenome Modifiers on Sex Chromosomes

##### Lysine Demethylase 6A (KDM6A)

**Figure 5:**
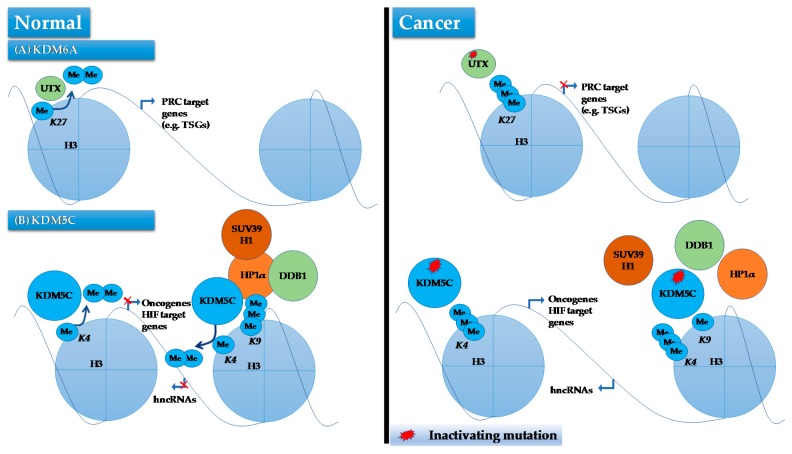
Functional consequences of KDM6A (or UTX) and KDM5C inactivation. (**A**) H3K27me3 is a suppressive marks associated with PRC target genes. UTX removes methyl groups from H3K27 and activates PRC target genes including multiple TSGs. Accordingly, loss of UTX in ccRCC contributes to trimethylation of H3K27, leading to inactivation of PRC target genes (e.g., TSGs) [27,144]; (**B**) KDM5C is a histone demethylase (HDM) that remove methyl groups from H3K4me3. H3K4me3 is a mark of active transcription and the removal of methyl group(s) by KDM5C results in suppression of expression of many genes including HIF target genes. Therefore KDM5C has been suggested to play a role in controlling hypoxia-driven gene expression. In addition, demethylation of H3K4me3 by KDM5C at heterochromatic sites is required for the recruitment of silencing complex consisted of SUV39H1, HP1α, CUL4 and DDB1, which promotes tri-methylation of H3K9 for heterochromatin assembly and silencing of heterochromatic non-coding RNAs (hncRNAs). Inactivation of KDM5C in ccRCC, leads to the overexpression of genes that contribute to tumorigenesis including hypoxia-regulated genes, and uncontrolled expression of hncRNAs, which in turn triggers heterochromatin dysregulation and genome instability [147].

*KDM6A* or UTX, located on the Xp11.2, was the first histone modifier identified as being a human cancer gene mutated and inactivated in ccRCC [27]. This gene encodes a protein that demethylates H3K27, and is mutated in 1% of ccRCCs [143]. To investigate the functional consequences of *KDM6A* mutations Haaften et al. expressed wild-type *KDM6A* in two cancer cell lines (KYSE-180 and KYSE-450) that were deficient for *KDM6A*, as well as in a cell line with wild-type *KMD6A* (NCI-H1299) [27]. Authors observed a significant increase in doubling time only in *KDM6A*^−/−^ cell lines compared to control cells transfected with an empty vector. Using microarray analysis, it was shown that genes associated with the H3K27 methylating polycomb complex were enriched among those that were differentially expressed following introduction of *KDM6A* in *KDM6A*^−/−^ cell lines, indicating that restoration of KDM6A expression may regulate expression of these genes by modulating H3K27 methylation. In line with these observations, ChIP-PCR analysis confirmed a significant decrease in H3K27me3 levels on two polycomb target genes *SOX1* and *PCDH19* upon ectopic expression of KDM6A in these cells. These findings have been validated and expanded by a recent study reporting that loss of KDM6A in bladder cancer cells results in dowregulation of polycomb repressive complex 2 (PRC2) target genes, in line with an antagonizing function between KDM6A and PRC2 (Figure 5) [27,144]. Of significance, KDM6A-deficient cells displayed a dependency on EZH2, a member of PRC2 that methylates H3K27, for proliferation, suggesting that inhibition of EZH2 may be an effective therapeutic approach against KDM6A-mutated tumors [144]. Future studies are warranted to examine such vulnerability in KDM6A deficient ccRCC.

##### Lysine Demethylase 5C (KDM5C)

*KDM5C* or *JARID1C* is another X-linked histone demethylase-coding gene, which is mutated in 7–9% of ccRCC tumors [28,29,30]. The protein encoded by *KDM5C* removes methyl groups from H3K4me3, a histone mark associated with actively transcribed chromatin [145], and this demethylation results in transcriptional inactivation of genes [143].

It has been noted that HIF stimulates the expression of KDM5C, which in turn functions to repress expression of several HIF target genes including *IGFBP3*, *DNAJC12* and *COL6A1* by demethylating H3K4me3 on their promoters [145,146]. This indicates that KDM5C acts as buffer to control hypoxia-driven gene expression levels following constitutive HIF expression [145]. Given that the constitutive activation of HIF pathway (due to *VHL* inactivation) is the main driver pathway of ccRCC, *KDM5C* seems to act as a TSG in ccRCC. In addition, *KDM5C* knockdown in *VHL*^−/−^ cell lines has been shown to significantly increase tumorigenesis in a xenograft model of RCC, thus adding further evidence for a tumor suppressive role of *KDM5C* in ccRCC [6,145]. The extent of this tumor suppressive activity has been expanded by a recent study showing that KDM5C deficiency results in genomic rearrangements and instability, leading to aggressive forms of ccRCC [147]. In this study, authors determined that KDM5C not only catalyzes demethylation of H3K4me3 but also binds broadly to chromatin domains characterized by H3K9me3, a transcriptionally suppressive mark enriched in heterochromatin. At heterochromatic sites, KDM5C forms a complex with SUV39H1, HP1α, cullin 4 (CUL4) and the complex adaptor protein DDB1, which is required for tri-methylation of H3K9 for heterochromatin assembly and silencing. When KDM5C mutated, the formation of this complex is impaired, leading to an unrestrained expression of heterochromatic noncoding RNAs (hncRNAs), which in turn triggers heterchromatin dysregulation and instability (Figure 5) [147]. Additionally, KDM5C mutated ccRCCs show deregulated expression of ncRNAs and increased genomic rearrangements compared to those without such mutations [147]. Parallel to this study, the same group has also suggested that KDM5C functions in DNA replication at early origins independent of its transcriptional-regulatory role. They have reported that KDM5C dictates the assembly of the pre-initiation complex, by helping pre-initiation proteins, CDC45 and PCNA, binding to the chromatin. The pre-initiation proteins binding to chromatin is achieved through the demethylase activity of H3K4me3 by KDM5C [148]. Although the whole panel of genes regulated by KDM5C is unknown, it is likely that KDM5C regulates a large number of genes as H3K4me3 is a common mark of transcriptional activity throughout the genome. Further investigations combining transcriptome profiling with high-resolution epigenome mapping in RCC can shed light on molecular mechanisms by which KDM5C contributes to ccRCC, and may lead to novel therapeutic interventions.

Interestingly, *KDM5C* and *KDM6A*, escape the X-inactivation, and have recently been identified among genes called ‘escape from X-inactivation tumor suppressors’ or EXITS genes [19]. Furthermore, the homologs of these two genes on chromosome Y, *KDM5D* and *KDM6C*, were found to be down-regulated due to loss of chromosome Y in 40% of male ccRCC patients [149]. Proteins coded by these Y-linked genes share over 80% of sequence similarly with products of their homologs on X-chromosome and have similar function, suggesting that their deficiency may contribute to ccRCC development [149] (Figure 2).

#### 3.1.3. Potential Clinical Significance of Mutations in Histone/Chromatin Modifiers

Recent studies have revealed complexity of ccRCC genomes, with mutations that are ubiquitous, shared and/or private [150]. Ubiquitous mutations are acquired in the early stage of tumorigenesis whereas private are those acquired later in the process and shared mutations are in between [114]. *VHL* and *PBRM1* mutations have been suggested as ubiquitous while mutations in *BAP1* and *SETD2* seem to be private and acquired later in the ccRCC development. Interestingly, in ccRCC, mutations in *BAP1* and *PBRM1* tend to be mutually exclusive [100,122,151]. Moreover, most mutations in *SETD2* and *KDM5C* also occur in a mutually exclusive manner [28,118]. Generally, if mutations in genes are exclusive it indicates that the genes function in the same pathway. However, many studies have suggested that *BAP1* and *PBRM1*, function in two different pathways as do *SETD2* and *KDM5C* [6,114,121,152]. In fact, *BAP1* mutations in tumors are associated with pathological features of aggressive disease including high Fuhrman grade [118,122,151,153], metastatic disease at presentation, worse cancer specific survival, instantaneous activation of mTOR signaling, and activation of pathways implicated in growth factors [118,122,151,153]. Similarly, ccRCC patients with *KDM5C* mutations manifest an aggressive form of the disease [147]. In contrast, in *PBRM1* mutated tumors, mTOR activation only occurs after a long latency period [95,118,122,151,153]. Moreover, results regarding the clinical significance of *PBRM1* or *SETD2* mutations are conflicting [118,122,151,153].

*BAP1* and *PBRM1* mutations are associated with characteristic and non-overlapping gene expression signatures, and outcomes for patients with ccRCCs mutated in *BAP1* or *PBRM1* are different [118]. As such, *BAP1* and *PBRM1* mutations have been suggestive of two different molecular subtypes of ccRCC with different biology and outcomes. On the other hand, interestingly, there appear to be a positive genetic interaction among *PBRM1* and *SETD2*; a meta-analysis show that the frequency of *SETD2* mutations in ccRCC is twice as high in tumors with *PBRM1* mutations [100]. This suggests that a selective pressure to mutate *SETD2* may exist in PBRM1-mutated tumors, although the molecular basis remains unknown. Curiously, however, both BAF180 (encoded by *PBRM1*) and SETD2 are involved in epigenetic regulation, one as a reader (BAF180) and the other as a writer (SETD2) [114,121].

Another study highlighted that the loss of *BAP1* and *PBRM1* are likely events that are required in both ccRCC development and progression to metastasis [154]. As such, they showed that status of BAP1 and PBRM1 protein expression is concordant between patient-matched primary and metastatic lesions. Moreover, intratumoral heterogeneity in metastatic tumors was minimal at protein expression of BAP1 and PBRM1. This suggests that results from examining expression of these two proteins in primary tumors may be applicable to patient-matched metastatic samples. However, additional studies including larger number of samples are required to verify these findings.

#### 3.1.4. Other Histone Modifications

Acetylation that usually occurs at lysine residues of histones is considered as the most frequent histone modification [42,155]. Acetylation neutralizes the basic charges on histone tails thereby reducing their affinity towards DNA. This altering interactions between histones of adjacent nucleosomes as well as between histones and other regulatory proteins creates new binding surfaces [92,156,157]. Acetylation usually results in the activation of transcription, and deacetylation is associated with suppression of gene expression [92,156,157]. Little is known about the dysregulation of histone marks in ccRCC; however, it has been suggested that histone H3K9 acetylation is reduced in 85% of the ccRCCs compared to corresponding non-neoplastic tissue [158]. Notably, treatment with Depsipeptide, a HDAC inhibitor, reduced cell proliferation and induced apoptosis in dose- and time-dependent manners in RCC cell lines, while increasing H3K9 acetylation, p21(WAF/CIP1) expression and phosphorylation of Bcl2 [158]. Furthermore, Mosashvilli et al. has reported that total H3Ac levels were inversely correlated with pT-stage, distant metastasis, Fuhrman grading and RCC progression. Findings from these studies suggest that elevation of H3 acetylation through treatment with HDACs may potentially be a therapeutic approach for ccRCC [159]. Wang et al. (2013) showed that a HAT of the MYST family, MYST1 (also known as hMOF) that acetylates histone H4K16 was frequently downregulated in 90% (19/21) of ccRCCs. Moreover, the H4K16 acetylation levels were downregulated correlating with downregulation of hMOF in ccRCC samples [160].

### 3.2. DNA Methylation

Precise DNA methylation patterns are crucial for genomic stability, parental imprinting, and importantly, control of gene expression [161,162]. Abnormalities in methylation patterns can cause instability and/or altered expression of the genome resulting in tumorigenesis and cancer [34]. Generally, methylation of the DNA occurs at the cytosine bases that are located at 5′ of a guanosine in human genome [163,164]. When happens at a CpG island, a 500 bp < region of DNA with a GC content of at least 50%, hypermethylation of CpGs within a promoter causes inactivation or silencing of TSG. In contrast, hypomethylation of the promoter CpGs of proto-oncogenes causes their activation [6,35,85,165]. Addition of methyl groups on DNA is carried out by DNA methyltrasferase (DNMT) 1, DNMT3A and DNMT3B which are highly expressed in cancers [166]. DNMTs and other methyl-CpG-binding proteins (e.g., MBD2, MBD3 and MeCP2) recruit histone modifiers (e.g., histone deacetylase and chromatin remodelling complexes) to the methylated promoter region [166,167,168], and deacetylates histones 3 and 4 and stimulates chromatin condensation and gene silencing [6,169]. Furthermore, many studies have pointed out the occurrence of CpGs in other genomic regions that are essential for gene regulation including enhancers, repressors and regulatory regions within gene body [6,170,171,172,173,174].

Extensive hypermethylation of CpG islands, a pattern known as CpG island methylator phenotype (CIMP), has been observed in 20% of ccRCC tumors [6,30,175]. CIMP-associated tumors present with aggressive phenotypes, such as poor overall survival rates and patient outcome, and an elevated activity of anaerobic glycolysis pathway, which provide energy to tumor cells [176,177,178,179].

A spectrum of genes has been identified to be silenced due to promoter hypermethylation in ccRCC [6,34,35,42,180,181,182]. These genes are involved in essential cancer-related pathways including pro-apoptotic genes (e.g., *APAF-1*, *LRRC3B*); negative regulators of the cell cycle (e.g., *KILLIN*, *RASSF1*, *p16*, *BTG3*); key regulators of Wnt and TGF-β pathways, and DNA mismatch repair genes (e.g., MSH2) [42]. However, an interesting observation is the methylation-driven dysregulation of Keap1/Nrf2 pathway in ccRCC. Previous studies have shown that constitutively active Nrf2 pathway, due to loss of Keap1 (Kelch-like erythroid-derived Cap-n-Collar Homology (ECH)-associated protein-1), results in acute kidney injury, chronic kidney disease, cancer [183,184], and activation of Phase II enzymes [185]. These activated Phase II enzymes confer neoplastic cells resistance to radio- and chemotherapies and growth and survival advantages during their transformation and progression [186]. Although genetic alterations of the Keap1/Nrf2 pathway were reported only in a small fraction of ccRCCs (6.6%) [132], tumor-specific DNA methylation of the *KEAP1* gene promoter has been detected in 48.6% of the tumors [186]. This abnormal methylation pattern was not observed in other histological subtypes of RCC. Moreover, they found a direct correlation with mRNA levels using an in vitro 5-azacytidine treatment. In addition to this, authors analyzed 481 ccRCC TCGA data sets independently and showed that KEAP1 methylation is associated with tumor staging, grading and overall survival of patients. These findings were the first to link epigenetic silencing of *KEAP1* promoter to KEAP1 expression changes in ccRCCs and corroborate the driver role of Keap1/Nrf2 axis deregulation in ccRCC. Several other genes have been identified affected by abnormal hypermethylation in ccRCC either by analyzing DNA methylation of tumor samples or cell line models treated with methylation inhibitors such as 5-aza-2-deoxycytidine in combination with gene expression profiling. Catalogues of aberrantly methylated silenced genes in RCC, their respective function and expression changes can be found in other publications [6,34,35,42,180,181,182].

Most of the RCC methylome studies have concentrated on methylation at the promoter regions; however, it has been argued that epigenetic changes at enhancer regions can also influence tumorigenesis. In line with this, high-resolution methylation analysis interrogating 1.3 million CpG loci across the RCC genome has revealed an enrichment of aberrantly hypermethylated loci at kidney-specific enhancer regions, which are associated with the H3K4me1 mark. More importantly, an enrichment of hypermethylated regions has been reported for binding sites of *AP2a*, *AHR*, *HAIRY*, *ARNT*, and *HIF1* transcription factors. Silencing of these factors contributes to dysregulated hypoxia signaling pathway in RCC. This finding uncovered a probable link between constitutive activation of the HIF pathway and widespread epigenetic modifications in RCC [173]. Likewise, *JAG1*, was aberrantly hypomethylated in ccRCCs, resulting in overexpression of the *JAG1*, a ligand in NOTCH signaling. The loss of DNA methylation on the *JAG1* locus coincided with H3K4me1 chromatin modification at the active enhancer region [174]. Using high-resolution assays, additional studies uncovered that transcribed regions in gene body are highly methylated and the degree of methylation is positively correlated with level of expression proposing that transcribed region of gene methylation may be crucial in transcriptional regulation [69,187,188,189,190,191]. As such, additional studies focusing on other regulatory elements including methylation at gene body, enhancers and repressors are ongoing [6].

Another form of epigenetic marks that could regulate gene expression is hydroxymethyl cytosine, which occurs by oxidation of 5 methylated cytosine (5hmC) [192,193]. This process is catalyzed by a family of methylcytosine dioxygenase ten eleven translocation (TET) proteins (TET1, TET2 and TET3) [194]. Genome-wide 5hmC levels are suggested to be markedly reduced in ccRCC tissue compared to adjacent, non-malignant tissue [195]. However, the precise mechanism and role in tumorigenesis is yet to be determined. Nevertheless, it appears that 5hmC levels could be used as a potential biomarker for ccRCC [6].

### 3.3. Long Non-Coding RNA (LncRNA)

Gene expression can be regulated via control of the final protein concentration through transcriptional and post-transcriptional (but pre-translational) regulation of expression by noncoding RNAs. Once considered as transcriptional noise, lncRNAs, have attracted much attention during the past few years as potentially new and crucial layer of biological regulation [196,197]. LncRNAs are non-protein coding RNAs of more than 200 bps size which are found in every branch of life [197,198]. LncRNAs are of two types; intergenic and intragenic and are expressed in a tissue-specific manner and many undergo through 5′ end capping and 3′ end polyadenylation similar to mRNAs [35]. LncRNAs have been implicated in a range of developmental processes (reviewed in Refs. [199,200]) and diseases including cancer, but knowledge about their mechanism of action is still at its infancy [196]. In cancer, overexpression, downregulation or mutation of lncRNA genes has been observed (reviewed in Ref. [201]). In RCC, deregulation of several lncRNAs have been reported with potential impacts on RNA-protein interaction networks that function in splicing, transport, localization, and processing of RNA [202]. However, an oncogenic or tumor suppressor activity has only been validated for a few of these lncRNAs, as we will discuss below (Table 1) [202,203,204].

#### 3.3.1. Oncogenic LncRNA in RCC

Metastasis-associated lung adenocarcinoma transcript 1 (MALAT1), is located on chromosome 11q13, and is one of the most studied lncRNA that is expressed constitutively in normal tissues [35,205]. In multiple cancers such as non-small cell lung carcinoma, hepatocellular carcinoma, lung cancer, cervical cancer, pancreatic carcinoma as well as ccRCC, MALAT1 is overexpressed and acts as an oncogene [35,205,206] by enhancing cell growth, tumorigenesis and metastasis through modulation of ERK/MAPK pathway [35,207]. The expression level of MALAT1 was higher in ccRCC tissues compared to adjacent non-tumor tissues, and knocking down MALAT1 expression decreased ccRCC proliferation, migration and invasion, supporting its involvement in ccRCC tumorigenesis, as reported recently [206,208,209].

HOX transcript antisense RNA (HOTAIR) is another well-studied lncRNA, which acts as an oncogene and interacts with EZH2, a subunit of PRC2. PRC2 is a HMT that trimethylates H3K27 resulting in silencing of various TSGs involved in cancer progression of many cancers such as breast, gastric, colorectal, and cervical cancer cells [35,208]. HOTAIR functions as a scaffold for the assembly and targeting of PRC2 and LSD1/CoREST/REST complexes to the HOXD locus thereby the transcription through modulating H3K27 methylation and H3K4 demethylation [210,211]. In fact, HOTAIR physically interacts with PRC2 and LSD1 complexes via its 5′ and 3′ domains [205,206]. The knockdown of HOTAIR can inhibit proliferation, migration, and invasion of cancer cells. Increased expression of *HOTAIR* in RCC has been reported, and inhibition of *HOTAIR* expression has been shown to decrease cell proliferation and invasion in vitro and to suppress growth of xenograft RCC tumors in vivo [207,212]. Furthermore, *HOTAIR* suppression via siRNA induced cell cycle arrest at the G0/G1 phase and resulted in weakening of the recruitment and binding abilities of EZH2. Likewise, the silencing of *HOTAIR* and the associated changes in H3K27 methylation activated transcriptional state of cell cycle-related genes [201]. HOTAIR has recently been implicated in the metastasis of RCC. This function has been connected to a dual regulatory role of HOTAIR on chromatin state at different genomic loci. Xia et al. showed that suppression of HOTAIR by siRNAs induced expression of EZH2 target gene *PCDHB5*, and simultaneously reduced expression of histone demethylase JMJD3 and its target gene *Snai1* [213].

H19 is located at the Igf2/H19 imprinted gene cluster at the telomeric end of chromosome 11, and encodes a maternally imprinted lncRNA. H19 is completely repressed in most human tissues and only expressed during the embryonic period [214,215,216]. HIF1α has been shown to trigger H19 expression under hypoxia. H19 is involved in mesenchymal-to-epithelial transition (MET) and EMT by influencing the function of EZH2, β-catenin and E-cadherin [216]. Overexpression of H19 has been observed in many cancers including breast, bladder, ovarian and gastric cancer and RCC [208,216]. KO of H19 reduced proliferation, migration and invasion thereby reducing tumorigenesis of ccRCC [217]. Higher expression of H19 is associated with significantly shorter overall survival (OS) [208].

SPRY4 intronic transcript 1 (SPRY4-IT1) gene, located on chromosome 5, specifically, in the second intron of the *SPRY4* gene, produces a lncRNA, which is proposed to be involved in cell growth and apoptosis [218]. *SPRY4-IT1* acts as an oncogene and was found to be elevated significantly in several types of cancers including ccRCC [219,220]. Suppression of this lncRNA resulted in inhibition of proliferation and invasion, and elevated rates of apoptosis [218,221]. Furthermore, it has also been shown to play a role in EMT by regulating E-cadherin and vimentin expression in glioma [222]. Higher expression levels of SPRY4-IT1 were associated with histological grade, tumor stage, distant metastasis and poorer OS in ccRCC [219].

Recently, another lncRNA, long intergenic non-coding RNA 152 (LINC00152), was found to be up-regulated in RCC tissues [223]. Expression of LINC00152 was found to be positively correlated with lymph node metastasis, higher TNM stage, and poor OS in RCC patients. Inhibition of LICN00152 reduced cell proliferation. Interestingly, LINC00152 was found to bind EZH2, LSD1 and methylated histone H3 at lysine 27 (H3K27me3) and epigenetically suppressed expression of TSG p16 [223].

#### 3.3.2. Tumor Suppressive LncRNAs in RCC

LncRNAs could potentially have tumor suppressive roles. LncRNA maternally expressed gene 3 (*MEG3* or *Glt2*), located on chromosome 14, is ubiquitously expressed and stimulates many physiological functions in human embryonic stem cell and mature human tissue. These functions are related to neurogenesis and insulin synthesis [208,224,225,226]. Furthermore, it acts as TSG via activation of p53 in several human cancers such as lung cancer and glioma [227,228,229]. In RCC, it is markedly downregulated in vivo and in vitro [230]. Re-expression of *MEG3* in RCC cells have shown to elevate apoptosis via activation of the intrinsic mitochondrial pathway [230]. However, there is no information about possible associations of MEG3 expression levels with clinic-pathological parameters in RCC [208].

The Growth Arrest Specific 5 (*GAS5*) gene is located on chromosome 1 and is involved in p53- and Baculoviral IAP repeat-containing protein 3 (cIAP2)-dependent apoptosis. It also inhibits cell proliferation through p21, CDK6 and cyclin D1 [231,232,233]. *GAS5* is downregulated, acts as a TSG in RCC and suppresses proliferation, invasion and migration of ccRCC cells [232]; however, no correlation between GAS5 levels and clinicopathological parameters of RCC has been reported [35,208].

LncRNA Cell adhesion molecule 1 antisense transcript 1 (CADM1-AS1) gene is located at the chromosome 11, precisely, at the antisense direction of the exon coding for *CADM1*. *CADM1* encodes a membrane protein required for cell-to-cell interactions, regulates cell cycle, apoptosis and differentiation [234] and acts as TSG in several types of cancers [235]. *CADM-AS1* levels were found to be lower in ccRCC samples than normal tissues, and lower expression levels were significantly associated with worse OS and advanced clinical stage. *CADM1-AS1* expression in ccRCC cells resulted in lower migration and apoptosis and growth rates [234].

The above-mentioned lncRNAs are a few examples of potential oncogenic or tumor suppressive lncRNAs in RCC; however, the majority of them need further validation by independent studies that characterize their function with further details [35,208].

## 4. Epigenetic Therapy for RCC

### 4.1. Histone Deacetylase (HDACs) Inhibitors

Generally, many cancers show overexpression of HDACs and a genome-wide reduction of histone acetylation, which is associated with reduced expression of genes including key TSGs [236,237]. In fact, HDAC-1 and -2, are overexpressed in 50% of RCC cases and are suggested to silence essential TSG. Thus, several studies are being carried out to verify the efficacy of some HDAC inhibitors (e.g., valproic acid) for their anti-tumor activity in RCC [238,239].

Touma et al. has shown that the combination of HDAC inhibitors (class of hydroxamic acids), retinoic acid and trichostatin A (TSA), reduced proliferation of human RCC cell lines in vitro and inhibited tumor growth while increasing apoptosis in a xenograft model in vivo [240]. Tumor growth inhibitory effect was through the enhancement of retinoic acid pathway and was stronger when compared to monotherapy with either of HDAC inhibitor or 13-*cis*-retinoic acid (CRA) alone. Treatment with MS-275, a benzamine derivative HDAC inhibitor, in combination with CRA showed stronger reduction of tumor growth compared to single monotherapy with either of the drugs [241]. In addition, two interesting observations were made during the study: firstly; treatment withdrawal caused tumor reoccurrence compared to continuously-treated mice, hence confirming the treatment effect; and secondly, an induction of retinoic acid receptor β2 (RARβ2) was observed, suggesting that HDAC inhibitors might reverse retinoid resistance.

EMT is one of the key features that occurs during tumorigenesis of ccRCC and other cancers. One study showed that treatment with TSA prevents TGF-β1-induced EMT in cultured human renal proximal tubular epithelial cells [242]. TGF-β1 co-treatment with TSA significantly prevented TGF-β1-induced downregulation of E-cadherin and upregulation of collagen type I [242], suggesting that HDAC inhibitors may potentially be used in therapeutic approaches against EMT in ccRCC.

HDACs can, in part, regulate cell cycle and apoptosis through their effects on pRB and p53 [243]. Hence, HDACs inhibitor (e.g., LBH589), when used in combination with rapamycin (an mTORC1 inhibitor), showed significant suppression of tumor growth and angiogenesis in vitro and in vivo in RCC cell lines and xenografts [243]. Another study showed upregulation of p53 and VHL expression and downregulated HIF1α and VEGF using TSA [244]. TSA also showed anti-angiogenesis effects in vitro and in vivo. Moreover, the two previous studies have shown that the HDACs inhibitors destabilize and reduce HIF1α levels via proteasomal degradation as proposed by Jeong et al. [245]. Initially, this effect was thought to be regulated by hyperacetylation and increased expression of VHL and p53 [244]. However, same effect was observed in *VHL*^−/−^ RCC and p53^−/−^ cell lines suggesting that the observed proteasomal degradation of HIF1α is independent of VHL and p53 and does not require the ubiquitin system [246].

Another HDAC inhibitor, panobinostat (hydroxamic acid class), has been reported to decrease hypoxia-induced cisplatin resistance in lung cancer cells by promoting HIFα destabilization [247]. These findings suggest that application of HDAC inhibitors in *VHL* deficient ccRCC could potentially render tumors chemosensitive by HIFα destabilization countering the resistance that exists in ccRCC currently. Interestingly, ritonavir, a HIV protease inhibitor, has been reported to act synergistically with panobinostat to increase histone acetylation and inhibit RCC growth in xenografts [248].

Despite these promising results from preclinical studies, outcomes of clinical trials of HDAC inhibitors were not interesting due to very low response rates and severe adverse effects [238,239,249,250], suggesting that monotherapy with these drugs do not improve patients outcome. Considering combinational therapy, vorinostat, (a hydroxamic acid class HDAC inhibitor) synergized with the anticancer activity of temsirolimus (inhibitor of mTOR pathway), both in a panel of RCC cell lines in vitro and in two xenograft models in vivo [251]. As such, cell viability and clonogenic survival was reduced while apoptosis was increased in the in vitro models. Moreover, tumor burden and angiogenesis was reduced markedly due to a decrease in HIF2α expression and vessel density in vivo. In addition, combination treatment resulted in a potent of survivin, concomitant to the induction of apoptosis and reduced proliferation of tumor cells [251].

A unique study used combination of a HDAC inhibitor, romidepsin, and a methyltransferase inhibitor, decitabine, to test their anti-cancerous activity on stage IV ccRCC and triple-negative breast cancer (TNBC) cell lines [252]. Stage IV ccRCC and TNBC are metastatic solid tumors that are aggressive and mostly drug resistant. Combination of romidepsin and decitabine showed synergistic effects on the inhibition of cell growth and induction of apoptosis above levels of monotherapy of each drug. This synergistic effect was due to reexpression of a TSG called secreted frizzled-related protein one (sFRP1). This study showed that combinatorial treatment with romidepsin and decitabine in drug resistant tumors is a promising treatment strategy for advanced ccRCC and TNBC [252].

Deleterious *BAP1* mutations are associated with deficiency in melanocytic differentiation in uveal melanoma (UM) and cancer metastasis. Landreville et al. [130] reported that treatment of cultured UM cells with HDAC inhibitor VPA induces differentiation, cell-cycle arrest, and a switch to a differentiated gene expression profile characteristic of melanocytes. Furthermore treatment with VPA showed an anti-growth effect on UM tumors in vivo. Moreover, RNAi-mediated knockdown of *BAP1* in UM cells induced a marked increase in H2A ubiquitination and treatment with VPA resulted in a substantial reduction of histone H2A ubiquitination levels in BAP1-deficient cells. Thus, HDACs inhibitors might be effective for treatment of RCC in patients with *BAP1* mutations as they might have the ability to reverse H2A ubiquitylation, which is associated with *BAP1* mutated ccRCC [130]. Taken together, these results suggest that combined treatment approaches involving HDAC inhibitors and current available targeted therapies might be promising to be considered in the future clinical trials.

### 4.2. Histone Methyltransferases (HMTs) and Histone Demethylases (HDMTs) Inhibitors

EZH2 is the core catalytic subunit of PRC2 that acts as a HMT by trimethylating H3K27, which is involved in silencing of several TSGs. Silencing of EZH2 by its inhibitor S-adenosylhomocysteine hydrolase inhibitor 3-Deazaneplanocin A (DZNep) significantly inhibited migration and invasion of RCC cell lines while up-regulating the expression of E-cadherin in vitro. Also, DZNep inhibited tumor growth, and prolonged survival in xenograft models of RCC [253,254,255].

As mentioned above, *SETD2* (a HMT) is required for H3K36 trimethylation and this H3K36me3 is frequently lost in SETD2-mutated ccRCC. Importantly, H3K36me3 facilitates the expression of RRM2, a ribonucleotide reductase subunit essential for production of dNTPs. Notably, RRM2 is also regulated by a second pathway in which CDK promotes RRM2 degradation. The activity of CDK is controlled by WEE1, and upon WEE1 inhibition, hyperactive CDK promotes RRM2 degradation. Hence, concomitant deficiency of H3K36me3 and inhibition of WEE1 results in lower amounts of RRM2, which triggers unscheduled mitotic entry leading to loss of genome integrity, which in turn results in replication stress and aberrant origin firing leading to cell cycle arrest and apoptosis [256]. This concept has been validated and used by Pfister et al. to design a synthetic lethal approach for H3K36me-deficient tumors. They showed that a WEE1 inhibitor (AZD1775) reduced size of *SETD2*-deficient (or H3K36me3 deficient) tumors in xenograft mouse models highlighting the promising effect of targeting *SETD2* mutated/H3K36me3 deficient tumors cells by WEE1 inhibitors [256].

### 4.3. DNA Methyltransferases (DNMT) Inhibitors

Numerous genes and pathways related to ccRCC are deregulated due to promoter hypermethylation. Demethylating agents or DNMT inhibitors (e.g., decitabine or azacitidine) have shown to reduce methylation at global genomic level including gene promoters [6], suggesting that reversing abnormal promoter methylation could be a potential therapeutic strategy. Treatments with DNA methylation inhibitors have shown promising results for haematological neoplasia such as acute myeloid leukaemia and chronic myelomonocytic leukaemia [257]. Likewise, treatment with azacitidine has suppressed cell proliferation in all 15 RCC cell lines tested in a recent study [258]. Moreover, decitabine has been shown to inhibit the growth of a human metastatic RCC cell line in a xenograft model, by restoring *connexin 32* (*Cx32*) gene expression [259]. Despite these potentially interesting observations, no large-scale trial for treatment of RCC with DNA methylation inhibitors is currently underway [6].

## 5. Conclusions and Emerging Concepts

The fact that mutations in histone modifying and chromatin remodeler genes (such as *PBRM1*, *BAP1*, *SETD2*, *KDM5C* and *KDM6A*) are frequent in RCC, and that the products of these genes can regulate multiple signaling pathways (e.g., mTOR, p53 and pRB–E2F), highlight the driving roles of genomic abnormalities in RCC. This has well been documented with predisposition to RCC in families with germline mutations in *PBRM1* and *BAP1*. Likewise, emerging data propose a possible role for mutations of the X-linked epigenome modifier genes *KDM5C* and *KDM6A* in predominance of RCC in male patients [18,19,149,260,261]. KDM5C and KDM6A are among the six EXITS genes with a significantly higher frequency of mutations in tumors (including ccRCC) of male patients than those from female patients [18,19,260]. As these genes escape X-inactivation, males would require only a single deleterious mutation whereas females would require two mutations for the complete loss of proteins coded by these genes. Therefore, mutations of these genes may explain, at least in part, a fraction of higher incidence of RCC in males. Further investigations involving functional experiments are needed to examine these possibilities, and to screen additional TGS genes on sex chromosome that may be involved in the sex-biased prevalence of RCC.

The widespread alterations of epigenetic patterns in RCC may open a new avenue for development of novel therapies involving epigenetic machineries. Intriguingly, drugs targeting the epigenetic system are either in clinical use or in clinical trials for different cancers; however, strategies that combine these novel epigenetic therapies with conventional therapies for RCC, i.e., those targeting angiogenesis or the mTOR pathway, are still at infancy. Although there is a possibility of increased adverse effects associated with combination therapy, carefully choosing specific combinations based on genomic profiles of tumors can reduce this risk, and may provide a solution to overcome therapy resistance in RCC patients. Supporting this, Hsieh et al. have reported that patients with *KDM5C* mutated tumors experience a prolonged response to sunitinib treatment as *KDM5C* mutations were associated with longer first-line progression free survival [262]. While promising, additional genomic studies involving large number of samples are required to validate these associations. Given the fast advancements in epigenome mapping technologies, we expect that in the near future, high-resolution epigenome landscapes of tumors as well as experimental models perturbed for epigenome modifiers will generate invaluable information about their mechanisms of action. Similarly, approaches that can investigate chromatin accessibility alterations without using information about DNA methylation or histone marks, as described by Becket et al. [263], could uncover novel regulators of epigenetic and gene expression patterns [263].

## Figures and Tables

**Table 1:** Newly identified lncRNAs along with their location, expression and abnormal function in ccRCC. EGF, embryonic growth factor; EMT, epithelial-mesenchymal transition; MET, mesenchymal–epithelial transition; TSG, tumor suppressor gene; LSD1, lysine (K)-specific demethylase 1A (LSD1) or KDM1A; Ch, Chromosome.

Name	Location	Expression Levels	Effect on Tumor	Abnormal Function in ccRCC
MALAT1	Ch 11	Up regulated	Oncogene	PRC2 control. Activating ERK/MAPK pathway to cause tumorigenesis. Also involved in EMT transition via E-cadherin recovery and β-catenin downregulation
HOTAIR	Ch 12	Up regulated	Oncogene	PRC2 control. Plays a dual regulatory role in chromatin state by affecting both histone methylation, via elevating EZH2 (of PCR2) target gene *PCDHB5* levels, and demethylation, via downregulating demethylase JMJD3 and its target gene *Snai1* expression
H19	Ch 11	Up regulated	Oncogene	EGF involved in MET and EMT by influencing the function of EZH2, β-catenin and E-cadherin
SPRY4-IT1	Ch 5, second intron of SPRY4	Up regulated	Oncogene	Inhibition of MAPK pathway and a role in EMT by regulating E-cadherin and vimentin expression
LINC00152	Ch 2	Up regulated	Oncogene	Binds EZH2, LSD1 and H3K27me3. Promotes tumorigenesis by epigenetically repressing p16 expression
MEG3	Ch 14	Down regulated	Tumor suppressor	Stimulation of apoptosis, acts as TSG via activation of p53
GAS5	Ch 1	Down regulated	Tumor suppressor	Stimulation of apoptosis via p53 and cIAP2 and inhibition of cell proliferation via p21, CDK6 and cyclin D1
CADM1-AS1	Ch 11, antisense of the exon of CADM1	Down regulated	Tumor suppressor	Cell to cell interaction, regulates cell cycle, apoptosis and differentiation. Higher levels in ccRCC reduces migration and tumor growth rates.
lnc-BMP2-2, lnc-CPN2-1, lnc-FZD1-2, lnc-ITPR2-3, lnc-SLC30A4-1, & lnc-SPAM1-6	-	Up regulated	-	RNA-protein networks that function in splicing, transport, localization and processing of RNA.
lnc-ACACA-1, lnc-FOXG1-2, lnc-LCP2-2, lnc-RP3-368B9, & lnc-TTC34-3	-	Down regulated	-	
